# Cuerpos de mora en sedimento urinario de una paciente con enfermedad renal crónica

**DOI:** 10.1515/almed-2019-0039

**Published:** 2020-04-27

**Authors:** Carlos Martínez-Figueroa, Karen Cortés-Sarabia, Hilda Guadalupe Catalán-Nájera, Micaela Martínez-Alarcón

**Affiliations:** Servicio de urgencias, Laboratorio clínico, Clínica Hospital ISSSTE, Iguala, Guerrero, Mexico; Laboratorio de Inmunobiología y Diagnóstico Molecular, Facultad de Ciencias Químico Biológicas, UAGro, Chilpancingo, Guerrero, Mexico; Heroico Colegio Militar 3, Centro, Iguala de la Independencia, Gro, 40000, Iguala, Mexico

**Keywords:** cuerpos de mora, enfermedad de Fabry, enfermedad renal crónica, sedimento urinario

## Abstract

**Objetivos:**

La Enfermedad de Fabry es un trastorno hereditario ocasionado por una mutación en el gen que codifica para la α galactosidasa A, lo que ocasiona la acumulación de glicoesfingolípidos en distintos órganos. El diagnóstico oportuno de esta enfermedad es vital para comenzar el tratamiento y evitar la disfunción orgánica debido a la acumulación de lípidos. Por lo anterior, diversos investigadores han analizado la búsqueda de cuerpos y células de mora como una nueva alternativa del diagnóstico, de lo cual a continuación se describe un caso.

**Caso clínico:**

Paciente femenino de la tercera edad, sin antecedentes de enfermedades crónicas, y con síntomas compatibles con infección del tracto urinario (disuria, dolor pélvico y polaquiuria). En base a los resultados de laboratorio, se le diagnostico anemia acompañada de una enfermedad renal crónica. En el examen químico del uroanálisis se presentó una microhematuria, acompañada de proteinuria, mismo que fue observado en el sedimento urinario, adicionalmente se observaron partículas lipídicas compatibles con cuerpos de mora.

**Conclusiones:**

La identificación de los cuerpos y células de mora en el sedimento urinario es una herramienta sencilla que puede ayudar en el diagnóstico de la enfermedad de Fabry, lo que puede coadyuvar en el tratamiento oportuno de reemplazo enzimático para reducir las afecciones sistémicas.

## Introducción

La enfermedad de Fabry (EF) es un trastorno sistémico ligado al cromosoma X, ocasionado por una mutación en el gen *GAL*, lo que se asocia con deficiencias en la enzima α galactosidasa A y que genera la acumulación progresiva de glicoesfingolípidos y globotriaosilceramida (GL-3) que se acumulan en el endotelio del corazón, sistema nervioso central y riñones [1], [2]. Las manifestaciones clínicas más severas se presentan en varones hemicigotos, entre las que se encuentran afecciones cardiovasculares, enfermedad renal crónica terminal y eventos cerebrovasculares. En contraste, las mujeres heterocigotas, pueden presentar una sintomatología leve o cursar de forma asintomática [3]. El diagnóstico de EF se realiza mediante la determinación de la concentración de la enzima α galactosidasa A en plasma. Sin embargo, estudios recientes han demostrado que en el sedimento urinario pueden detectarse células y cuerpos de mora [2]. A continuación se describe un caso de una paciente con enfermedad de Fabry que presenta estas estructuras.

## Caso clínico

**Figura 1:**
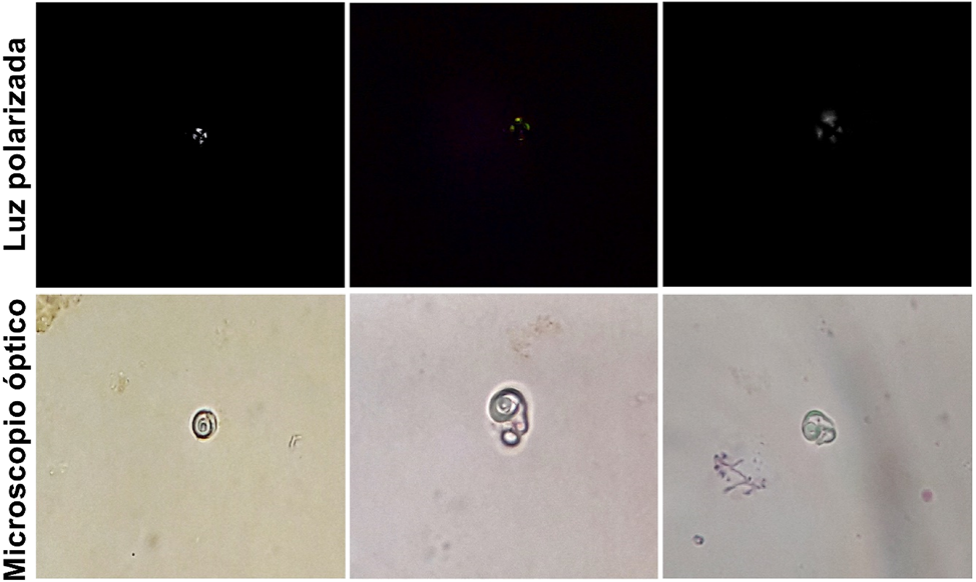
Observación de cuerpos de mora en sedimento urinario. Parte superior: cruz de malta de los cuerpos de mora observada con microscopía de luz polarizada 40×. Parte inferior: estructuras lipídicas con morfología atípica observadas con microscopía de campo claro 40×.

Describimos una paciente femenina de 88 años de edad, llegó al servicio de urgencias por presentar dolor pélvico, disuria y polaquiuria por lo que fue diagnosticada con una presuntiva infección del tracto urinario. La paciente no contaba con un historial clínico, por lo que se le solicitaron estudios de laboratorio para su evaluación. En la citometría hemática (Swelab Swelab Alfa Basic, Boule Diagnostics), la paciente presentó una hemoglobina de 8,9 g/dL (12–15 g/dL), hematocrito 28% (38–47%), leucocitos de 14,14 × 10^3^/mm^3^ (5–10 × 10^3^/mm^3^) y plaquetas de 573 × 10^3^/mm^3^ (150–400 10^3^/mm^3^). El estudio bioquímico sanguíneo (RX daytona, RANDOX) mostró una glucosa de 76 mg/dL (70–110 mg/dL), urea de 97 mg/dL (10–50 mg/dL), creatinina de 1,6 mg/dL (0,6–1,3 mg/dL), sodio de 153 mmol/L (135–148 mmol/L), potasio de 4,2 mmol/L (3,5–5,3 mmol/L) y cloruro de 95 mmol/L (98–107 mmol/L). La tasa de filtración glomerular (TFG) era de 30,5 mL/min/1,73 m^2^. El examen químico urinario mostró una densidad de 1.010, pH de 8,5, proteínas trazas, hemoglobina (25 hematíes/µL) y esterasa leucocitaria (15 leucocitos/µL). En el sedimento de la muestra se confirmó la hematuria (4–6 eritrocitos/campo) y adicionalmente se observaron unos pocos cilindros granulares, así como escasas bacterias y células escamosas y cilíndricas. También se observaron estructuras de origen lipídico con apariencia de espiral, lo que fue confirmado por la presencia de una cruz de malta en luz polarizada. Estas estructuras fueron compatibles con cuerpos de mora patognomónicos de la EF (Figura 1), por lo que se realizó la conjetura de que el origen de la enfermedad renal crónica era una nefropatía de Fabry.

## Discusión

Los pacientes con EF, comúnmente desarrollan una nefropatía, siendo los primeros signos la albuminuria durante la niñez y proteinuria a la edad de 20–30 años, mientras que a partir de los 50 años los pacientes ya presentan una enfermedad renal crónica terminal [4]. Su diagnóstico temprano es de vital importancia para iniciar el reemplazo enzimático y evitar la progresión de las afecciones. Sin embargo, algunos casos atípicos de la EF no presentan un cuadro clínico habitual o las alteraciones se manifiestan en un órgano, lo que conlleva an una progresión de los padecimientos cardiacos y renales [2], [4]. La paciente descrita en el caso no presentaba los síntomas habituales, pero mostró un deterioro de la función renal lo que fue evidente por los altos niveles de urea y de creatinina, y la disminución de tasa de filtración glomerular. Estos valores clasifican en un estadio G3b (enfermedad renal crónica) según las recomendaciones KDIGO [5]. La paciente no presentaba antecedentes de diabetes mellitus tipo 1 o 2, que es una de las principales causas la enfermedad renal. La citometría hemática reveló anemia, lo que puede ser secundario a la enfermedad renal crónica. Las estructuras observadas en el sedimento urinario, identificadas como cuerpos de mora, provienen de las células de mora, que son células epiteliales del túbulo contorneado distal con globotriaosilceramida acumulado en forma de micelas con cuerpos laminares en su interior formando una espiral. Estas partículas se encuentran frecuentemente en pacientes con EF [6]. La importancia de estas estructuras en el diagnóstico de la EF ha sido previamente descrita, Selvarajah et al. [7], que reportaron que la detección de los cuerpos de mora tienen una sensibilidad y especificidad del 100% en el diagnóstico de la EF, observándolas tanto en pacientes con y sin nefropatía. En base a lo anterior, se ha propuesto a las células de mora como una alternativa en el diagnóstico de la EF cuando no se dispone de métodos como la cuantificación de la enzima y se sospecha del padecimiento de esta enfermedad. En conclusión, se recomienda realizar un análisis exhaustivo del sedimento en la búsqueda de estos tipos celulares para corroborar el diagnóstico e iniciar de manera oportuna el tratamiento [6], [8], [9].

### Cinco puntos de aprendizaje

– La EF se genera por la deficiencia de enzima α galactosidasa A.
– La acumulación de glicoesfingolípidos a nivel renal condicionan la aparición de una enfermedad renal crónica.
– Los cuerpos de mora tienen una especificidad y sensibilidad del 100% en el diagnóstico de la EF.
– Los cuerpos de mora son estructuras lípidicas con un cuerpo laminar en su interior en forma de espiral.
– Los cuerpos y células de mora se pueden presentar antes de que el paciente presente la nefropatía de Fabry.
